# Comparison of nutritional, bioactive potential and antioxidant properties of *Saba senegalensis* fruit pulps from five regions of Burkina Faso

**DOI:** 10.3389/fnut.2024.1358968

**Published:** 2024-04-04

**Authors:** Salamata Tiendrebeogo, Clarisse Sidbewendé Compaoré, Raymond Poussian Barry, Edwige Bahanla Oboulbiga, Mamoudou Hama Dicko

**Affiliations:** ^1^Département de Technologie Alimentaire (DTA), Institut de Recherche en Sciences Appliquées et Technologies (IRSAT), Centre National de la Recherche Scientifique et Technologique (CNRST), Ouagadougou, Burkina Faso; ^2^Laboratoire de Biotechnologie, Technologie Alimentaire et Nutrition (LABIOTAN), Département of Biochimie et Microbiologie (DBM), University Joseph KI-ZERBO, Ouagadougou, Burkina Faso

**Keywords:** *Saba senegalensis*, pulp, bioactive compounds, antioxidant, region “Mansfelder Land”

## Abstract

**Introduction:**

The fruit of *Saba senegalensis* plays an important role in household nutrition. It is an important source of sweet carbohydrates, minerals, vitamin C, provitamin A and has many biological properties. It is also of economical importance and employment for rural populations, through the processing of fruit. Unfortunately, the lack of exhaustive data on the composition and properties of the fruit and its derivatives limits processing and marketing. The species is widespread in different climatic zones.

**Methods:**

Therefore, its composition and biological properties may vary, offering a variety of processing products to meet the specific nutritional needs. This study aimed to characterize the bioactive potential and antioxidant properties of fruit pulps of *S. senegalensis* in order to increase its value-added processing. Pulp samples of fruits were sampled from five regions of Burkina Faso, namely the Cascades, Sud-Ouest, Boucle du Mouhoun, Nord and Centre-Sud regions.

**Results and Discussion:**

Qualitative analysis showed the presence of alkaloids, saponins, terpenoids and steroids, anthocyanins and tannins. Quantitative analyses showed a significant variation in phenolics, tannins, lycopene, vitamin C, β-carotene and antioxidant activity among samples. However, this variation was not region-dependent. Indeed, some fruits from same region showed both the highest and lowest values for the assessed parameters. Fruits from regions of Centre-Sud and Sud-Ouest and displayed the highest and lowest levels of total phenolics (877.48 and 1142.33 mg GAE/100 g) and tannins (42.38 and 55.64 mg TAE/100 g), respectively. The high potential of *S. senegalensis* fruits pulp in nutritional and bioactive compounds, and antioxidant properties recorded in this study suggests that they can be used as a dietary supplement or in the formulation of energy foods and nutraceutical containing foods.

## 1 Introduction

In recent decades, there has been a growing awareness of the importance of a diet rich in fruit and vegetables, as demonstrated by the declaration of the International Year of Fruit and Vegetables by the Food and Agriculture Organization of the United Nations (FAO) in 2021. In 2017, around 3.9 million deaths worldwide were attributable to insufficient consumption of fruit and vegetables (1). Therefore, the World Health Organisation (WHO) recommends daily consumption of fruits and vegetables for their beneficial effects on health and nutrition, as well as their role in a healthy, balanced diet and lifestyle (2). It is intended to contribute to the prevention of certain metabolic diseases such as cardiovascular pathologies, obesity, diabetes, neurodegenerative diseases, cancer, etc. (3, 4). Epidemiological studies have shown that a diet rich in fruit and vegetables were associated with a reduction in these metabolic diseases (5). Many constituents and oligo-elements in these foods, such as fiber, vitamins, minerals, polyphenols and antioxidants, play a protective role. Interestingly, fruits from *Saba senegalensis* may be candidates as source of several micronutrients.

*Saba senegalensis* is a wild liana that grows in the African savannahs and is known by various calls in different linguistic dialects, including *Weda* (in the *Mooré* language in Burkina Faso), *Zaban* (in the *Malinké* language in Mali) or madd fruit, magubo, Saba, etc. (in other languages). All parts (fruits, leaves and roots) of the plant are used as traditional remedies for many illnesses (6). Green fruits combat galactagogues and colic, and is an effective diuretic (7). The ripe fruits are anorectic, antiscorbutic, stimulant and tonic. The roots are used to treat female sterility. Macerated leaves are used against vomiting and stomach aches, latex against coughs and tuberculosis, and tendrils for baby care (6). Leaves and twigs are used in handicrafts to make dyes (6). The fruit of *S. senegalensis* has great therapeutic potential. Also, many previous studies have reported the high nutritional potential of its fruit. The fruit is an important source of nutrients, particularly vitamins (pro-vitamin A and ascorbic acid) dietary fiber and minerals such as potassium, magnesium and calcium (8). The presence of bioactive compounds such as phenolics compounds in *S. senegalensis* fruits has also been reported. These compounds could play an important role in the prevention and treatment of oxidative stress related diseases (9).

In Burkina Faso, *S. senegalensis* is distributed across all the country’s climatic zones, with high densities in the Sahelian and Soudanian zones. Despite its high potential in nutritive and bioactive compounds, its antioxidant properties and economic contribution, its processing remains limited. This is in part linked to seasonality and high perishability of its fruits. However, the lack of comprehensive data on the composition and antioxidant properties of the fruit and its by-products (pulp, hulls) limits the possibilities for exploiting the fruit. In Burkina Faso, previous studies on *S. senegalensis* have focused on the nutritional potential of the fruit (10, 11). Those relating to the bioactive compounds of the fruit and pulp are not exhaustive. Moreover, depending on the tree’s area of distribution, its composition and biological properties may vary. A better understanding of these aspects would make it possible to set up processing techniques for better valorization of *S. senegalensis* fruits, and to provide products enriched with bioactive compounds that will be accepted by consumers. The aim of this study was to characterize the bioactive potential and antioxidant properties of *S. senegalensis* fruit pulp collected in different localities of Burkina Faso, in order to provide database for its industrial processing.

## 2 Materials and methods

### 2.1 Collection sites and sampling

**Figure 1:**
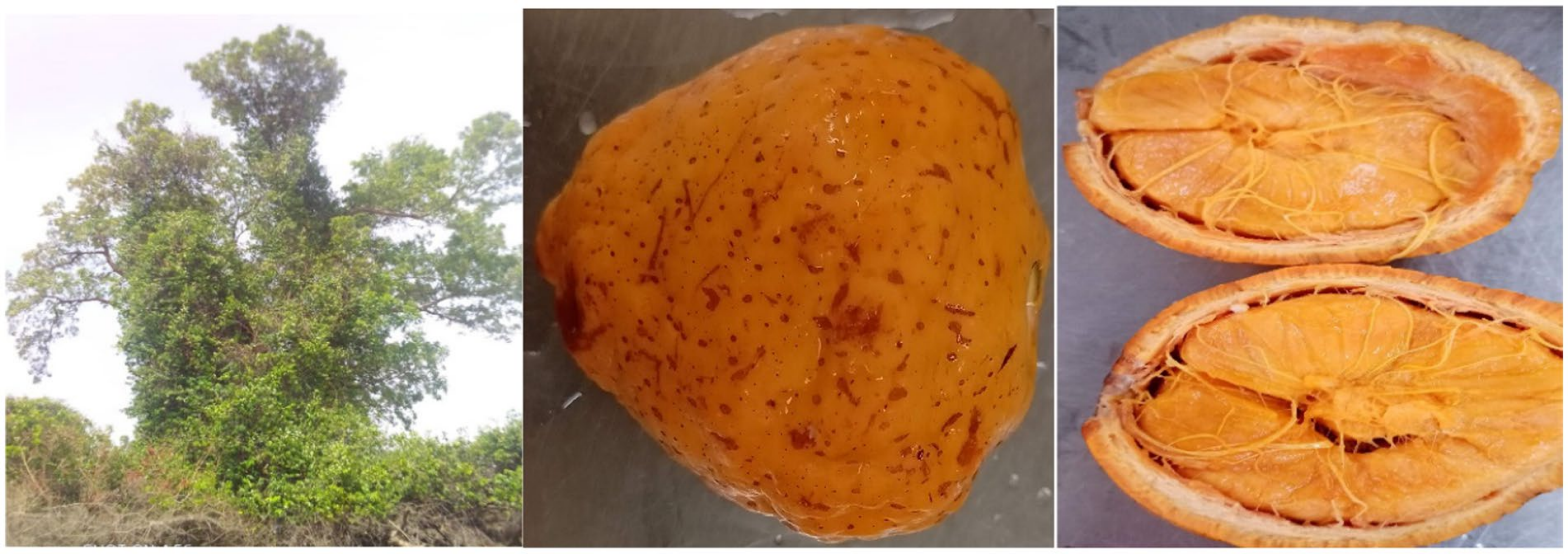
Tree, whole fruit and cross-section of the fruit showing the pulpy seeds for *Saba senegalensis* fruit.

**Figure 2:**
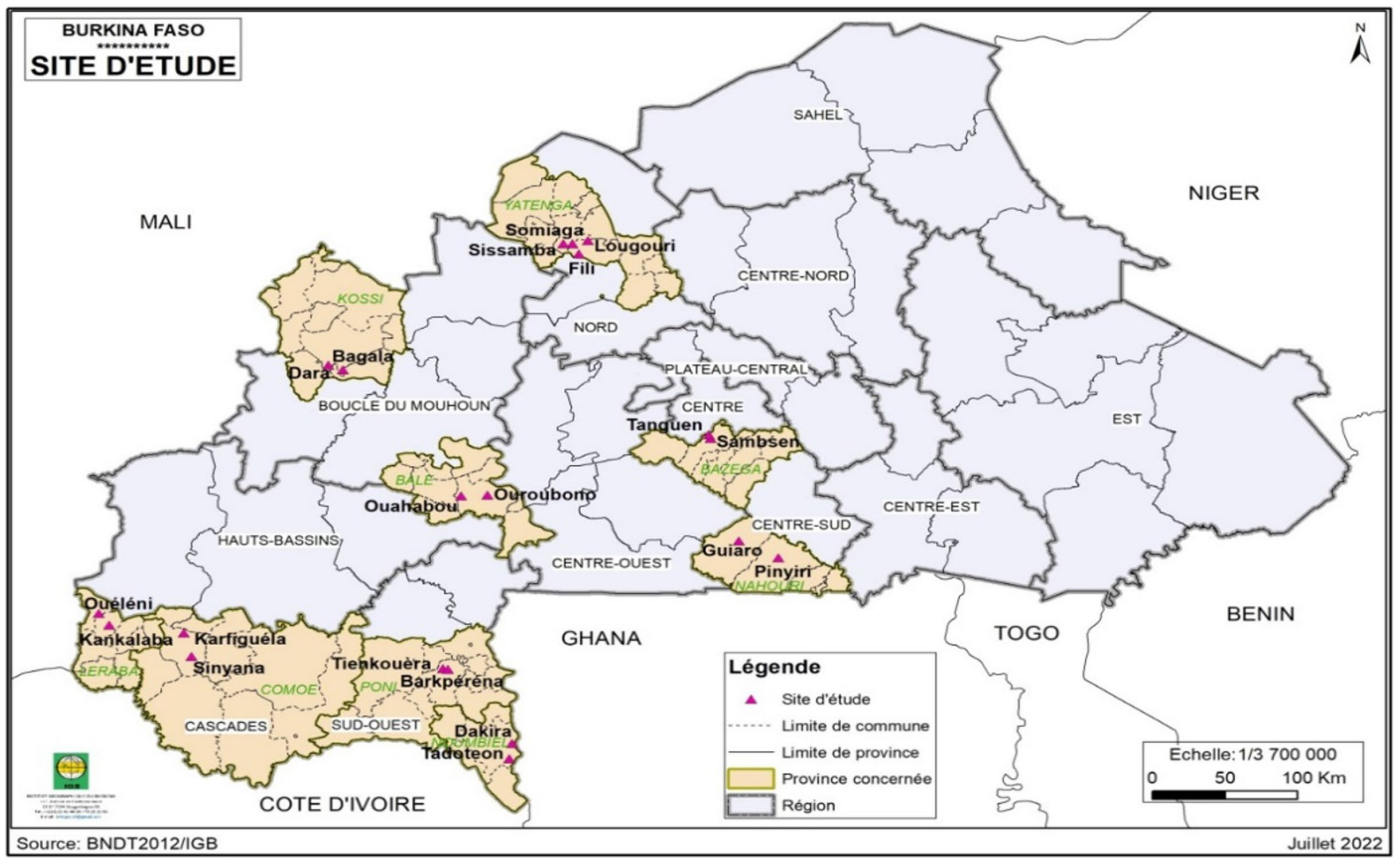
Map showing the sampling sites (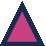).

*Saba senegalensis* fruits (Figure 1) used for the study were harvested from five regions of Burkina Faso. Four villages were identified in each region (Table 1). The collection sites (Figure 2) were chosen according to the fruit availability. Samples were collected between June and September 2021 in different regions according to the ripening time of the fruit.

**Table 1:** The main regions according to the different sampling villages for *Saba senegalensis* fruit.

Name of region	Villages
Cascades	Karfiguela, Sinyana, Kankalaba and Oueleni
Sud-Ouest	Dakira, Tadoteon, Barkperena and Tienkouera
Boucle du Mouhoun	Bagala, Dara, Ouahabou and Ouroubono
Nord	Sissamba, Sounkouissi, Fili and Lougouri
Centre-Sud	Guiaro Pinyiri, Sambsen and Tanguen

Sampling was carried out on batches of fruit picked at maturity on different trees with the help of local people and environmental agents during the fruiting period from one region to another. In each region, the collect took place in 4 villages (Table 1). Approximately 2–5 kg of fruits per tree were collected and 5 trees were selected per village. A total of 100 samples were collected. The fruit samples were transported to the Food Technology Department’s pilot work-station for processing. After sorting, a 2 kg of fruit sample were taken at random from each batch. The pulp extracted from the seeds were collected in jars and kept in the freezer at −18°C prior to analysis.

### 2.2 Methods

#### 2.2.1 Determination of biochemical composition of pulps

##### 2.2.1.1 Carotenoids and lycopene

Sample (100 mg of pulp) was homogenized with 5 mL of acetone/hexane (70/30) and the resulting mixture was stirred for 5 min and then centrifuged at 4500 rpm for 15 min. After extraction, the absorbances of the samples were read using a HELIOS EPSILON brand spectrophotometer at wavelengths of 453, 505, and 663 nm (12). The β-carotene and lycopene contents were expressed in μg/100 mg pulp, using the Formulas 1 and 2:


(1) $$\begin{array}{l} \mathrm{Lycopene}\;\left(\mathrm{mg}/100\;\mathrm{ml}\right)=-0.0458\;A_{663}+0.372\;A_{505}\\ \;–0.0806\;A_{453} \end{array}$$

(2) $$\begin{array}{l} \beta -\mathrm{carotène}\;\left(\mathrm{mg}/100\;\mathrm{ml}\right)=0.216\;A_{663}–0.304\;A_{505}\\ \;+0.452\;A_{453} \end{array}$$

where the underscore number of each A letter represents the wavelength.

##### 2.2.1.2 Ascorbic acid

The method used is based on the decolorization of 2,6-dichlorophenolindophenol (DCPIP) by ascorbic acid. For this, 50 μL of the extracts (50 mg/mL) were added to 150 μL of DCPIP (0.2 mM). The absorbance was read on a spectrophotometer at 515 nm against a blank consisting of 150 μL DCPIP and 50 μL water distilled. A calibration curve was plotted with ascorbic acid in the concentration range of 10–100 μg/mL. Ascorbic acid levels were expressed as μg ascorbic acid equivalent per 100 g of pulp (μg EAA/100 mg fresh pulp) (13).

#### 2.2.2 Analyses of other bioactive components and antioxidant properties

##### 2.2.2.1 Preparation of extracts

Extraction was made by maceration. An aliquot of 500 mg of pulp of fruit was mixed with 10 mL of ethanol (80%; v/v). The mixture was stirred for 24 h, then centrifuged at 4500 rpm for 30 min. The supernatant was collected and stored in the refrigerator at 4°C in dark prior to various analyses.

##### 2.2.2.2 Total phenolics

Their levels in pulp extracts were quantified spectrophotometrically (14) using the Folin–Ciocalteu Reagent (FCR). In each well of the plate, 25 μL of each extract was mixed with 125 μL of FCR (0.2 N). After homogenization by vortexing for 5 min, 100 μL of sodium carbonate (75 g/L) was added. The mixture was incubated for 2 h and the absorbance was read at 760 nm using against a blank. The total polyphenol content was quantified using gallic acid (0–10 mg/mL) as standard and results were expressed as mg GAE/100 g fresh pulp.

##### 2.2.2.3 Total flavonoids

The method described by Zhishen et al. (15) with a few modifications was used for their quantification in pulp samples. Each aliquot of 75 μL (in 50 mg/mL) of the sample was homogenized in 75 μL of AlCl_3_ (2%). After 10 min of incubation, absorbance was measured at 415 nm using a spectrophotometer. The total flavonoid content was determined using the calibration curve (0–10 mg/mL) and the results were expressed as mg quercetin (EQ) equivalent (mg EQ/100 g fresh pulp).

##### 2.2.2.4 Total tannins

They were determined according to the method proposed by CEE (16). Briefly, 20 uL of extract (1 mg/mL) to be determined was mixed with 100 μL of water to which was added 20 μL of ferric ammonium citrate (28% iron; 3.5 g/L) (24 h old) and 20 μL of ammonia (8 g/L). The absorbance of the solution was measured at 525 nm after 10 min against a blank (20 μL extract +120 μL water +20 μl ammonia). Tannic acid was used as standard. Results were expressed as mg tannic acid equivalent (TAE) per 100 mg pulp (mg TAE/100 mg fresh pulp).

##### 2.2.2.5 Phytates

They were determined as previously described by Gonçalves et al. (17). Phytates extraction was performed by mixing 250 mg of sample in 10 mL of 2.4% HCl for 3 h at room temperature with constant stirring. The samples were clarified by centrifugation at 6000 rpm for 20 min at room temperature 20. The supernatant was applied and eluted from an anion-exchange resin (Dowex1x8–400, Sigma Co.). The assay was performed with 2.0 mL of Wade reagent [0.03% (w/v) FeCl3 and 0.3% sulfosalicylic acid] and 3.0 mL of the eluted sample. The absorbance was read at 500 nm using phytic acid as standard (18).

##### 2.2.2.6 Other qualitative analyses

The analysis of the puls targeted the presence of some bioactive compounds such as saponins, cardiotonic glycosides, terpenoids and steroids. The presence of saponins was carried out using the method described by Yadav and Agarwala (19). For the assay, 1 mL of extract was added to 3 mL of distilled water. The mixture was shaken for 2 min. The presence of saponin was revealed by the formation of persistent foam. For the determination of cardiac glycosides, 2 mL of glacial acetic acid containing a few drops of 5% ferric chloride was added to 5 mL of extract. Subsequently, 1 mL of concentrated sulfuric acid was added to the resulting solution. The formation of a brown halo at the interface indicates the presence of cardiac glycosides (20). For terpenoid assay, 2.5 mL of the extract was added to 1 mL of chloroform. After homogenization, 1.5 mL of concentrated H_2_SO_4_ was added to the mixture. The presence of terpenoid compounds was revealed by the formation of a red-brown color at the interface (21). Steroids were detected by the Liebermann Burchard test as follows: 2 mL of extract dissolved in 2 mL of chloroform and 2 mL of acetic acid were added along the wall, followed by 2 mL of concentrated sulfuric acid. The change in color from purple to green indicates the presence of steroids (22).

### 2.3 Antioxidant properties

#### 2.3.1 Antiradical activity of DPPH

The free radical scavenging capacity of the extract was determined using the DPPH (2,2-diphenyl-1-picrylhydrazyl) radical with some modifications. A 100 μL quantity of extract was mixed with 200 μL of 0.2 mg/mL DPPH ethanoic solution. The mixture was incubated for 15 min at room temperature and absorbance read at 517 nm against a blank made with 100 μL extract and 200 μL methanol. The mixture was kept in the dark for 30 min and the absorbance was measured (23). The percentage inhibition (I) was calculated using Formula 3:


(3) $$\%\mathrm{DPPH}=\left[\left(\mathrm{Abs}\;T–\mathrm{Abs}\;E\right)/\mathrm{Abs}\;T\right]\times 100$$

% DPPH: percentage of inhibition

Abs T: Absorbance of control

Abs E: Test absorbance

The Ferric Reducing Antioxidant Power (FRAP) method was also used (23) to assess free radical scavenging capacity. It is based on the reduction of ferric ion (Fe^3+^) to ferrous ion (Fe^2+^). For the assay, to a test tube containing 0.5 mL of sample solution (50 mg/mL), 1.25 mL of phosphate buffer (0.2 M, pH 6.6) was added 1.25 mL of potassium hexacyanoferrate K_3_[Fe (CN)_6_] (1%, w/v in water). The mixture was heated at 50°C in a water bath for 30 min. An aliquot of 1.25 mL of trichloroacetic acid (0.1%) was then added and the mixture centrifuged at 2000 rpm for 10 min. To 125 μL of the supernatant, 125 μL of distilled water and 25 μL of freshly prepared 0.1% FeCl_3_ in water were added in 96-wells microplate. A blank without sample was prepared under the same conditions. The reading was taken at 700 nm against an ascorbic acid standard curve (200 mg/L in distilled water). The iron-reducing potential of the tomato samples was expressed in mmol ascorbic acid equivalent per gram of extract (mmol EAA/g fresh pulp).

### 2.4 Statistical analysis

All analyses were conducted in triplicate. Data were processed to derive descriptive statistic values (e.g., means, coefficient of variation and relative standard deviation). In effect, statistical analyses included Principal Component Analysis (PCA) and Analysis of Variance (ANOVA). The Tukey test was performed to determine the statistical differences between the samples with a 95% confidence interval, using the XLSTAT-Basic 2020.3 version software.

## 3 Results and discussion

### 3.1 Nutritional and bioactive potential of *Saba senegalensis* fruit pulps

Screening showed the presence of all targeted phytochemical compounds except the steroid. The presence of these phytonutrients in *S. senegalensis* fruit pulp could justify its use as a source of nutraceutics. These phytonutrients have numerous preventive and curative functions in animal and human physiology. Saponins may act as hypotensive and anti-hyperlipidemic compounds (24). In addition to their antimicrobial and pharmacological properties alkaloids may play a detoxifying and local anesthetic role (25). Similarly, the presence of terpenes could inhibit the absorption of cholesterol and bile acids, with appreciable effects on LDH-cholesterol levels (26). *S. senegalensis* fruit pulp could therefore play a role in combating oxidative stress and help to prevent related diseases. The results of the qualitative analyses are confirmed by those of the quantitative analyses, which showed that *S. senegalensis* fruit pulp is an important source of bioactive compounds.

Quantitative analysis of the nutritional and bioactive compounds of fruits pulps of *S. sengalensis* (Table 2) showed a significant variation in compounds according to fruits provenance with the exception of vitamin C. However, the variations recorded were not a function of climatic zone. This variation can be explained by several factors such as genetic, fruit storage and harvesting conditions.

**Table 2:** Overall content of phenolic compounds and antioxidant capacities of *S. senegalensis* fruit pulp from 5 regions of Burkina Faso (fresh pulp: FP).

Regions	Villages	Polyphenols (mg GAE/100 g)	Flavonoids (mg QE/100 g)	Vitamine C (mg/100 g)	Β-Carotene (mg/100 g)	Lycopene (mg/100 g)	Phytates (mg/100 g)	Tannins (mg TAE/100 g)
Cascades	**Karfiguela**	1113.21 ± 23.64^bcde^	38.69 ± 3.80^f^	46.26 ± 16.12^a^	2.09 ± 0.21^ab^	1.09 ± 0.03^d^	5.61 ± 0.34^cd^	46.35 ± 0.47^abcd^
	**Sinyana**	1139.01 ± 95.41^bcd^	39.94 ± 5.42^f^	54.66 ± 5.80^a^	2.09 ± 0.43^ab^	1.09 ± 0.06^d^	5.61 ± 0.67^cd^	44.0 ± 3.76^abcde^
	**Kankalaba**	1126.79 ± 49.91^bcde^	73.03 ± 1.02^cd^	55.12 ± 5.60^a^	0.91 ± 0.42^cd^	1.27 ± 0.1^abcd^	16.39 ± 2.3^a^	47.4 ± 10.38^abc^
	**Oueleni**	1190.34 ± 41.69^bc^	52.3 ± 7.54^e^	49.87 ± 5.09^a^	1.29 ± 0.48^abcd^	1.25 ± 0.13^abcd^	16.79 ± 2.1^a^	36.3 ± 4.65^bcdefg^
	**Average**	**1143.33**	**50.99**	**51.47**	**1.59**	**1.17**	**11.10**	**43.51**
Sud-Ouest	**Dakira**	902.73 ± 37.89^ef^	74.47 ± 9.05^cd^	57.41 ± 6.38^a^	1.72 ± 0.00^ab^	1.19 ± 0.01^abcd^	8.64 ± 0.25^bc^	20.01 ± 0.89^fg^
	**Tadoteon**	1493.42 ± 68.13^a^	91.87 ± 5.12^a^	66.41 ± 8.34^a^	1.72 ± 0.00^abc^	1.39 ± 0.2^a^	8.16 ± 0.52^bcd^	19.06 ± 0.33^g^
	**Barkperena**	941.02 ± 56.00^def^	72.79 ± 2.91^cd^	56.83 ± 6.16^a^	0.77 ± 0.00^cd^	1.35 ± 2.71^abc^	6.26 ± 0.82^bcd^	29.68 ± 8.87^defg^
	**Tienkouera**	877.48 ± 34.65^f^	45.12 ± 1.77^f^	57.24 ± 6.05^a^	1.67 ± 0.02^abcd^	1.15 ± 0.03^abcd^	5.71 ± 1.17^cd^	39.88 ± 9.66^abcde^
	**Average**	**1053.66**	**71.06**	**59.47**	**1.47**	**1.27**	**7.19**	**27.16**
Boucle Du Mouhoun	**Ouahabou**	1222.11 ± 262.88^b^	53.35 ± 0.55^e^	53.17 ± 6.05^a^	1.57 ± 0.01^abcd^	1.14 ± 0.01^bcd^	8.71 ± 1.32^bc^	28.24 ± 2.32^efg^
	**Ouroubono**	955.05 ± 15.89^def^	53.35 ± 0.16^e^	44.49 ± 16.88^a^	1.57 ± 0.00^abcd^	1.14 ± 0.00^bcd^	8.71 ± 0.66^bc^	27.95 ± 0.87^efg^
	**Bagala**	980.32 ± 2.33^cdef^	85.4 ± 1.93^b^	54.82 ± 5.24^a^	1.56 ± 0.1^abcd^	1.24 ± 0.03^abcd^	6.41 ± 0.82^bcd^	34.05 ± 0.53^cdefg^
	**Dara**	982.57 ± 60.01^cdef^	77.57 ± 1.23^c^	54.73 ± 5.93^a^	0.72 ± 0.16^d^	1.37 ± 0.06^ab^	7.31 ± 0.87^bcd^	34.15 ± 0.35^cdefg^
	**Average**	**1035.01**	**67.41**	**51.80**	**1.35**	**1.22**	**7.78**	**31.10**
Nord	**Sissamba**	831,04 ± 4,89^f^	56.87 ± 1.04^e^	42.91 ± 14.76^a^	1.47 ± 0.35^abcd^	1.37 ± 0.01^ab^	6.78 ± 0.32^bcd^	34.89 ± 3.59^cdefg^
	**Sounkouissi**	755.26 ± 26.43^f^	55.12 ± 0.72^e^	52.95 ± 5.23^a^	1.15 ± 0.41^bcd^	1.23 ± 0.06^abcd^	7.41 ± 0.47^bcd^	36.66 ± 12.53^bcdef^
	**Fili**	831.03 ± 11.20^f^	57.32 ± 1.20^e^	50.45 ± 5.31^a^	1.47 ± 0.70^abcd^	1.37 ± 0.02^ab^	6.79 ± 0.65^bcd^	34.15 ± 0.71^cdefg^
	**Lougouri**	877.47 ± 93.42^f^	51.74 ± 2.36^e^	53.81 ± 6.00^a^	1.15 ± 0.41^bcd^	1.21 ± 0.01^abcd^	4.24 ± 0.55^d^	46.15 ± 4.12^abcd^
	**Average**	**823.70**	**55.26**	**50.03**	**1.31**	**1.29**	**6.30**	**37.96**
Centre-Sud	**Guiaro**	906.8 ± 29.63^ef^	67.77 ± 1.46^d^	50.94 ± 18.73^a^	1.15 ± 0.04^bcd^	1.12 ± 0.00^cd^	8.14 ± 3.9^bcd^	55.64 ± 7.16^a^
	**Pinyiri**	899.47 ± 27.76^ef^	66.87 ± 0.42^d^	57.44 ± 6.14^a^	1.36 ± 0.18^abcd^	1.19 ± 0.01^abcd^	7.89 ± 1.3^bcd^	52.41 ± 1.43^ab^
	**Sambsen**	1214.78 ± 129.17^b^	73.47 ± 1.74^cd^	54.82 ± 5.24^a^	1.62 ± 0.07^abcd^	1.11 ± 0.01^cd^	9.94 ± 1.1^b^	42.39 ± 2.15^abcde^
	**Tanguen**	1163.45 ± 152.64^bcd^	60.7 ± 1.66^e^	54.35 ± 5.79^a^	2.16 ± 0.69^a^	1.34 ± 0.19^abc^	4.91 ± 0.12^cd^	42.38 ± 8.23^abcde^
	**Average**	**1046.12**	**67.20**	**54.39**	**1.57**	**1.19**	**7.72**	**48.20**
	***P*-value**	**0.00**	**0.00**	**0.52**	**0.00**	**0.00**	**0.00**	**0.00**
General average	**1020.17 ± 191.65**	**62.38 ± 14.58**	**53.43 ± 9.09**	**1.46 ± 0.48**	**1.23 ± 0.12**	**8.02 ± 5.27**	**37.59 ± 10.69**	

In the same column, the means with the same superscript letters are not significantly different at the probability threshold *p* ≤ 0.0001.The values in bold represent the means for each region and the *p*-value.

Ascorbic acid (vitamin C) content of *S. senegalensis* fruit pulp from the 20 villages ranged from 42.91 ± 14.76 mg/100 g for Sissamba in the region of Nord to 66.41 ± 8.34 mg/100 g for Tadoteon in the region of Sud-Ouest. The levels found in the study are much higher than the values of Boamponsem et al. (27), Gayen et al. (28), and Kouakoua (29) in Senegal, Ghana, and Côte d’Ivoire, respectively; who found vitamin C contents ranging from 16.40, 32.86 to 36.67 mg/100 g, respectively. On the other hand, they are lower than those of Noba et al. (30) and Yao et al. (11) with an average value of 15.11 mg/100 g to 27.80 mg/g in Burkina Faso, but within the range of those obtained by Nafan and Silue (31) from 34.8 to 67.5 mg/100 g. These results show that *S. senegalensis* fruits are a important source of vitamin C. Consumption of the fruit could be beneficial to health given the protective role and antioxidant power of vitamin C (4). It is well-known as essential for skin health as a critical factor for collagen biosynthesis because of its involvement as co-factor for synthesis of hydroxy-lysine and hydroxy-proline. This vitamin is known for its antioxidant properties, which protect the cells and tissues of the human body against free radicals and oxidative stress (32, 33).

Pro-vitamine A content, i.e., β-carotene varied from 0.72 ± 0.16 mg/100 g for Dara in the region of Boucle du Mouhoun to 2.16 ± 0.69 mg/100 g for Tanguen in the region of Centre-Sud. The β-carotene content of *S. sengalensis* fruit pulp is similar to those of Kini et al. (34), Boamponsem et al. (27), and Sarr et al. (7) from Burkina Faso, Senegal, and Ghana, respectively; and is close to that found by Kouakoua (29) in Côte d’Ivoire, whose value was 1.96 ± 0.03 mg/100 g in freeze-dried pulp. The high presence of β-carotene in the pulp could make it candidate to be used to fortify foods in pro-vitamine A to combat avitaminose.

Lycopene levels in *S. senegalensis* fruit pulp ranged from 1.39 ± 0.2 mg/100 g for Tadoteon in the region of Centre-Sud to 1.37 ± 0.06 mg/100 g for Dara in the region of Boucle du Mouhoun. However, fruits from the Nord region recorded the highest average value (1.29 mg/100 g) and the Cascades region the lowest one (1.17 mg/100 g). This variation in samples between villages and regions may be due to climatic conditions and the degree of ripening of the fruit. Indeed, environmental factors, such as a high temperature of the fruit pericarpe, decreased the lycopene content in the fruit skin and also in the pulp (35). Moreover, lycopene content increases with fruit ripening (35).

Variations in β-carotene and lycopene content can also depend on several factors such as variety, degree of ripeness and agronomic conditions (36).

### 3.2 Phenolics and antioxidant properties of *Saba senegalensis* fruits pulps

Total phenolic compounds and antioxidant activity (Table 2) varied significantly among villages, independently of collection area. This variation could therefore be due to factors such as genetic, ripening degree, fruit storage as well harvesting conditions.

Total phenolics content of the pulp ranged from 755.26 ± 26.43 mg GAE/100 g in fruits from Sounkouissi in the region of Nord to 1493.42 ± 68.13 mg GAE/100 g in Tadoteon in the region of Sud-Ouest. Total phenolics content recorded in this study are higher than those found by Kouakoua (29) in Côte d’Ivoire, which obtained an average value of 600.94 mg GAE/100 g with freeze-dried pulp. However, some of obtained values are within the range of those obtained by Lamien-Meda et al. (37), Noba et al. (30), and Yao et al. (11) in Burkina Faso ranging from 132.80 mg GAE/100 g, 630.00 mg GAE/100 g to 945.83 mg GAE/100 g and Boamponsem et al. (27) in Ghana, found 984.15 mg GAE/100 g. These results show that the fruit of *S. senegalensis* is a good source of natural antioxidants justifying its traditional use as fruit displaying cathartic effect. Consuming them as they are could prevent certain diseases, which are now a public health problem in Burkina Faso.

The highest flavonoid concentration was observed with sample from Tadoteon in the region of Sud-Ouest with an average of 91.87 ± 5.12 mg QE/100 g while the sample from Karfiguela in the of Cascades showed the lowest concentration at 38.69 ± 3.80 mg QE/100 g. The recorded values are lower than those of Baiyeri et al. (38) and Kouakoua (29) in Nigeria and in Côte d’Ivoire with a value of 24,650 ± 2,250 mg/100 g (i.e., 24.65 ± 2.25%) to 245.09 mg QE/100 g of freeze-dried pulp. However, studies of Lamien-Meda et al. (37) and Yao et al. (11) in Burkina Faso, have reported lower values, which were 5.30 mg/100 g to 39.60 mg/100 g, respectively. This difference in concentration with the literature can be explained by the climate, harvesting period, soil type, extraction method and analytical methods. Data on flavonoid content in *S. senegalensis* fruit pulp show very high levels, which vary according to village and region. The presence of flavonoids in the pulp is an advantage for consumer health since flavonoids protect blood vessels from cholesterol-related damage. They are also known for their antioxidant, anti-inflammatory, diuretic, and artery-protecting properties (39).

The highest tannin contents were recorded in samples from the Centre-Sud region (Guiaro and Pinyiri), ranging from 55.64 ± 7.16 mg TAE/100 g FP to 52.41 ± 1.43 mg TAE/100 g FP, with an average value of 48.21 mg TAE/100 g FP. The lowest values were found in the Sud-Ouest region (Tadoteon and Dakira), with mean values of 19.06 ± 0.33 mg TAE/100 g FP and 20.01 ± 0.89 mg TAE/100 g FP (27.16 mg TAE/100 g FP). However, our values are much lower than those reported by Diabagaté et al. (8) and Kouakoua (29) with levels ranging from 198.94 mg TAE/100 g to 356.10 mg TAE/100 g and Yao et al. (11) in Burkina Faso who found an average of 80.30 mg TAE/100 g. This difference may be linkedto extraction conditions, dosage method, climate, soil, ripening, and harvesting time. However, the astringent flavor of *S. senegalensis* could also be associated with the high tannin content. Although out tannins are often listed as anti-nutritional factor, notably for children, the presence of tannins in the fruit pulp could enable consumers to lower serum and liver total cholesterol levels (40).

Analysis of phytate content in pulp showed the highest values in the Sud-Ouest region (Oueleni) at 16.79 ± 2.10 mg/100 g. The lowest content was observed in the Nord region (Lougouri) with a value of 4.24 ± 0.55 mg/100 g. Statistical analysis showed a significant difference among pulp samples from different villages. The samples from the *Cascades* region recorded the highest average phytate value (11.10 mg/100 g) and also the lowest one (6.30 mg/100 g). Phytate levels are much lower than data reported in the pulp of *S. senegalensis* by Diabagaté et al. (8) in Côte d’Ivoire and Yao et al. (11) in Burkina Faso, which were 31.18 mg/100 g and 105.25 to 121.80 mg/100 g, respectively.

This difference depends on the nature of the soil, the climate, the extraction method, the ripening time, and the environment. Phytates (myoinositol hexaphosphates) have the particularity of chelating certain minerals by generating insoluble molecular complexes with divalent cations such as Ca^2+^, Fe^2+^, Zn^2+^, or Mg^2+^, which can impair their bioavailability and reduce their absorption and therefore their function (41). That is why phytic acid is considered as anti-nutritional factor.

**Figure 3:**
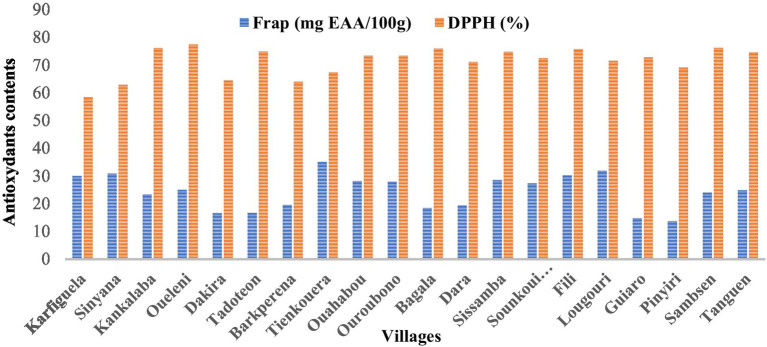
Comparison of antioxidant capacities of *S. senegalensis* fruit pulps in locations in Burkina Faso.

Antioxidant activity (Figure 3) shows a good capacity to reduce the DPPH radical in fruit samples from the region of Sud-Ouest, with the lowest average inhibition (67.63%), and the highest one in the region of Nord (73.62%). As a result, the pulp extract from the region of North has the highest antioxidant capacity compared to the other pulp extracts from the other regions. This was corroborated with ferric reducing activity power (FRAP) which was more marked with samples from the region of Nord with the highest mean value (29.52 mg EAA/100 g FP) and the lowest in the region of Centre-Sud (19.34 mg EAA/100 g FP), showing significant variation according to samples from different villages in the same region and among regions. The variation in the effective concentration of pulp extracts among villages could be explained by the variation in phenolic compound content, which is also influenced by the method of extraction and analysis. Previous studies have shown a correlation between the presence of phenolic compounds in an extract and its antioxidant activity (42). These analyses showed different antioxidant activities, which prove the influence of vegetation conditions, environment, and soil type on the polyphenol content and antioxidant potential of pulps (43).

### 3.3 PCA analyses performed base biochemical parameters

**Figure 4:**
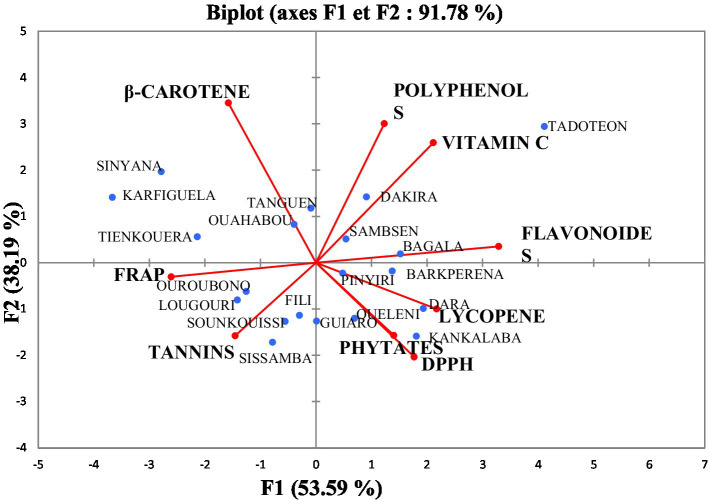
PCA of phenolic compounds and antioxidant activities of *S. senegalensis* fruit pulp in 20 locations in Burkina Faso.

Principal component analysis (PCA) performed based of all the pulp biochemical evaluated variables for the 5 regions (Figure 4). The analysis gives a total inertia percentage of 91.78%, i.e., 53.59% for F1 and 38.19% for F2 of the results. DPPH, phytates, vitamin C, polyphenols, β-carotene and Tannins were well associated on the F1 main axis. FRAP, lycopene, flavonoids parameters are the most represented on the F2 main axis. Significant positive correlations were recorded between total phenolics, flavonoids and vitamin C (Table 3). In addition, these three parameters were negatively correlated with iron reducing power and tannins. Phytates, lycopene and DPPH free radical scavenging capacity were positively correlated with each other, but negatively correlated with carotenes. Data (Table 3) show a very strong positive correlation of 100% between the polyphenols and the tannins, flavonoids and DPPH and FRAP and Phytates. Indeed polyphenols include tannins which are known to have high free radical scavenging properties. Investigations carried out on *Saba senegalensis* fruit pulps have shown that soil composition has an impact on the bioactive composition of pulps, particularly on polyphenol and tannin content. This results in a large variability in pulp composition from one region to another and from one village to another in the same region. Pulp composition also varies from tree to tree. The Sud-Ouest region (Tadoteon) has the highest levels of bioactive compounds in pulp.

**Table 3:** Pearson linear correlation matrix of the different variables.

Variables	Phenolics	Flavonoids	DPPH	FRAP	Vitamin C	β-Carotene	Lycopene	Phytates	Tanins
Phenolics	1								
Flavonoids	0.092	1							
DPPH	0.524	0.854	1						
FRAP	−0.642	−0.470	−0.774	1					
Vitamin C	0.533	−0.023	0.218	−0.286	1				
β-Carotene	0.189	0.194	0.197	−0.051	−0.409	1			
Lycopene	−0.414	−0.481	−0.521	0.095	−0.314	−0.374	1		
Phytates	−0.177	−0.651	−0.621	0.638	0.029	−0.160	0.135	1	
Tannins	0.615	0.569	0.751	−0.503	0.004	0.318	−0.415	−0.506	1

The PCA was used to group the different samples into four classes according to their biochemical characteristics:

- Group 1, constituted of samples from Sinyana, Karfiguela, Sinyana, Tanguen, Ouahabou and Tienkouera, is characterized by samples with good carotene levels. These results indicate a good source of natural food without resorting to chemical compounds that are not always harmless to health.
- Group 2 comprises samples from Sissamba, Sounkouissi, Ouroubono, Fili and Sissamba. These samples are characterized by higher tannin content and good FRAP activity. These compounds make it possible to fight against certain diseases and can be used as food supplements and medicines in the food industry.
- Group 3 is made up of Tadoteon, Dakira, Sambsen and Bagala, samples characterized by high levels of phenolic compounds and vitamin C. The fruits of these villages could be used as a food supplement to fight against certain avitaminoses.
- Group 4, constituted of samples from Pinyiri, Barkperena, Oueleni, Kankalaba, Guiaro and Dara, includes samples with high levels of carotenes, lycopenes and DPPH anti-free radical activity. The samples are a potential source of antioxidants. Therefore, the preservation of these bioactive compounds during processing and storage is important in order to be able to use them as beneficial elements for health.

## 4 Conclusion

The study revealed a variation in biochemical parameters studied, depending on *Saba senegalensis* fruit. This variation was not linked to the harvesting zone, but could be explained by genetic factors, fruit maturity and sample processing conditions. In addition, the study revealed that *S. senegalensis* fruit pulps are potential source of bioactive compounds, including free radical scaveniging molecules. Four sample chemotypes were therefore identified on the basis of their biochemical characteristics. These different chemotypes are of great interest to the food industry, manufacturing products enriched with micronutrients and bioactive compounds to help combat malnutrition and various metabolic diseases. Also, the fruit pulp is an interesting source that can be alternative local food product to achieve a satisfactory and balanced diet. The introduction of technologies to ennhance the economical and nutritional value of the pulp to increase daily intake of fruits and vegetables. The variability of its biochemical composition could mean that, depending on the quality of the end product required, fruit from different regions could be used without having to resort to chemical compounds that are not always harmless to health.

## Data availability statement

The original contributions presented in the study are included in the article/supplementary material, further inquiries can be directed to the corresponding author.

## Author contributions

ST: Conceptualization, Data curation, Formal analysis, Funding acquisition, Investigation, Methodology, Project administration, Resources, Software, Supervision, Validation, Visualization, Writing – original draft, Writing – review & editing. CC: Conceptualization, Data curation, Formal analysis, Funding acquisition, Investigation, Methodology, Project administration, Resources, Software, Supervision, Validation, Visualization, Writing – original draft, Writing – review & editing. RB: Writing – review & editing. EO: Writing – review & editing. MH: Conceptualization, Data curation, Formal analysis, Funding acquisition, Investigation, Methodology, Project administration, Resources, Software, Supervision, Validation, Visualization, Writing – original draft, Writing – review & editing.
